# Purification and Structural Analyses of Sulfated Polysaccharides from Low-Value Sea Cucumber *Stichopus naso* and Anticoagulant Activities of Its Oligosaccharides

**DOI:** 10.3390/md22060265

**Published:** 2024-06-08

**Authors:** Lige Cui, Huifang Sun, Xiaolei Shang, Jing Wen, Pengfei Li, Shengtao Yang, Linxia Chen, Xiangyang Huang, Haoyang Li, Ronghua Yin, Jinhua Zhao

**Affiliations:** 1 School of Pharmaceutical Sciences, South-Central Minzu University, Wuhan 430074, China; clg0407@163.com (L.C.); sunhuifang2021@outlook.com (H.S.); 15969709010@163.com (X.S.); 13708322361@163.com (P.L.); 18485566396@163.com (S.Y.); clx2870452496@163.com (L.C.); hxy0703123@163.com (X.H.); 202021151043@mail.scuec.edu.cn (H.L.); zhaojhscu@163.com (J.Z.); 2 School of Biology and Agriculture, Shaoguan University, Shaoguan 512005, China; jw82123@126.com

**Keywords:** *Stichopus naso*, polysaccharides, chemical depolymerization, well-defined oligosaccharides, anticoagulation

## Abstract

Three polysaccharides (SnNG, SnFS and SnFG) were purified from the body wall of *Stichopus naso*. The physicochemical properties, including monosaccharide composition, molecular weight, sulfate content, and optical rotation, were analyzed, confirming that SnFS and SnFG are sulfated polysaccharides commonly found in sea cucumbers. The highly regular structure {3)-L-Fuc_2S_-(α1,}_n_ of SnFS was determined via a detailed NMR analysis of its oxidative degradation product. By employing β-elimination depolymerization of SnFG, tri-, penta-, octa-, hendeca-, tetradeca-, and heptadeca-saccharides were obtained from the low-molecular-weight product. Their well-defined structures confirmed that SnFG possessed the backbone of {*D*-GalNAc_4S6S_-β(1,4)-*D*-GlcA}, and each GlcA residue was branched with Fuc_2S4S_. SnFS and SnFG are both structurally the simplest version of natural fucan sulfate and fucosylated glycosaminoglycan, facilitating the application of low-value sea cucumbers *S. naso*. Bioactivity assays showed that SnFG and its derived oligosaccharides exhibited potent anticoagulation and intrinsic factor Xase (iXase) inhibition. Moreover, a comparative analysis with the series of oligosaccharides solely branched with Fuc_3S4S_ showed that in oligosaccharides with lower degrees of polymerization, such as octasaccharides, Fuc_2S4S_ led to a greater increase in APTT prolongation and iXase inhibition. As the degree of polymerization increases, the influence from the sulfation pattern diminishes, until it is overshadowed by the effects of molecular weight.

## 1. Introduction

Fucosylated glycosaminoglycan (FG), also known as fucosylated chondroitin sulfate, is a glycosaminoglycan derivative with numerous fucosyl branches. Till now, this unique polysaccharide has been found widely and exclusively in sea cucumbers [1,2]. FGs from different species share the common backbone of {3)-D-GalNAc-(β1,4)-D-GlcA-(β1,}, whereas the branches vary in a species-specific manner regarding the sulfation pattern and glycosylation of fucosyl groups. For example, the fucose branches of FGs from *Stichopus variegatus* and *Massinium magnum* carry sulfate esters at O-2 and O-4 sites (Fuc_2S4S_) and O-3 and O-4 sites (Fuc_3S4S_), respectively [3,4]. Three types of branches, Fuc_2S4S_, Fuc_3S4S_, and Fuc_4S_ (only sulfated at the O-4 of fucose), were clarified in FG from *Apostichopus japonicus* [5]. In addition, studies in recent years have also found that fucose branches could be glycosylated by Gal or GalNAc to heterodisaccharide branches. The D-Gal-(α1,2)-L-Fuc types of branches have only been found in FG from sea cucumbers *Thelenota anana* [6]. D-GalNAc-(α1,2)-L-Fuc branches were reported in several FGs from sea cucumbers, including *Acaudina molpadioides*, *Holothuria nobilis*, *Ludwigothurea grisea*, and *Phyllophorella kohkutiensis*, whereas the sites and proportions of sulfation vary in different species [7,8,9,10]. Except branching from the O-3 of GlcA residues, the Fuc branch attached to the O-6 of GalNAc in the backbone was also suggested in FG from *Cucumaria frondosa* [11]. These variations constitute the structural diversity and complexity of the FG family.

In addition to its intriguing structure, FG has attracted increasing attention because of its broad spectrum of therapeutic properties, such as anticoagulation [12,13,14], anti-tumor [15,16], antiviral [17,18], and intestinal microbiota regulation [19,20]. Among them, its anticoagulant activity has been studied the most in depth, including structure–activity relationships and molecular mechanisms [14,21,22]. Low-molecular-weight FG and its oligosaccharides could potently and selectively inhibit intrinsic factor Xase complexes (iXase, VIIIa-IXa-PL-Ca^2+^ complex) [23]. Structure–activity relationship analyses of its wide range of pharmacological activities have revealed that the molecular mass and sulfate content are two common and key activity-influencing factors [24,25,26].

More generally, FG with simple repeating units is more valuable for accurate structure–activity relationship analyses. To date, structural units with predominantly Fuc_3S4S_ branches of FG have been found in Holothuriidae, including the sea cucumbers *M. magnum*, *Pearsonothuria graeffei*, *Bohadschia argus*, *Actinopyga miliaris*, and *Holothuria fuscopunctata* [4,27,28,29,30]. FG that is only branched by Fuc_2S4S_ was reported in the sea cucumbers *Isostichopus badionotus*, *Stichopus horrens*, *Stichopus chloronotus* and *Stichopus herrmanni* (formerly known as *S. variegatus*), belonging to two genera, *Isostichopus* and *Stichopus*, of the family Stichopodidae [26,27,31].

Besides FG, fucan sulfate (FS) is another common polysaccharide in sea cucumbers. FSs from the sea cucumbers *S. horrens*, *S. chloronotus* and *S. herrmanni* coincidentally share the common and highly regular structure of {3)-L-Fuc_2S_-(α1,} [32,33,34]. Another two simple repeating units of 4)-L-Fuc_3S_-(α1, and 4)-L-Fuc_2S_-(α1, were reported in *Holothuria fuscopunctata*, *Bohadschia argus* and *Thelenota anana*, respectively [32,35]. FS has garnered considerable attention in recent years due to its significant biological properties [1].

**Figure 1:**
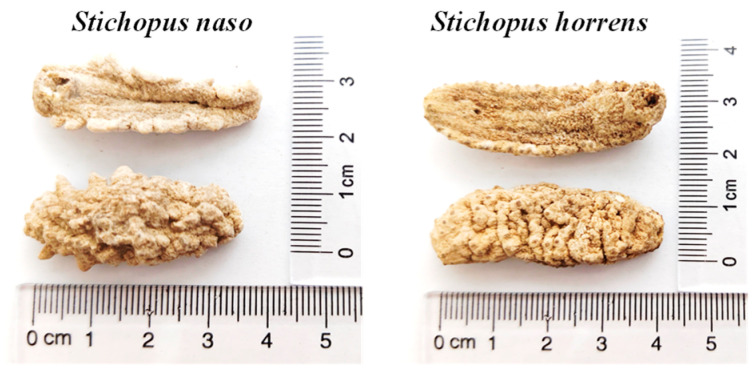
Processed specimens of *S. naso* and *S. horrens*.

*Stichopus naso* is a low-value sea cucumber of the genus *Stichopus*, widely distributed throughout the Indo-Pacific. It continues to be confused with *S. horrens* and traded under the same common name of “Huang Yu Shen” in China, as it is usually similar in size and appearance to *S. horrens*. In fact, prominent lateral papillae on the ventral margins should be more evident in the processed specimens of *S. naso* (Figure 1) [36].

Till now, little has been known about the structure and activity of *S. naso*-derived polysaccharides. Does FG or FS derived from *S. naso* have a highly regular repeat structure, like other species of the genus *Stichopus*? Herein, we extracted and purified three polysaccharides, including FG, FS, and neutral glucan (NG), from the body walls of *S. naso* (designated as SnFG, SnFS and SnNG, respectively). Their physicochemical properties, including monosaccharide composition, molecular weight, optical rotation, and sulfate content, were further analyzed. Moreover, peroxide depolymerization was employed for SnFS to obtain its low-molecular-weight product, thereby characterizing the structure of the prototype. A glycosidic-bond-selective depolymerization method, β-elimination depolymerization, was carried out for SnFG. Combined with six oligosaccharides purified from its low-molecular-weight product, the structure of SnFG was confirmed for the first time. The anticoagulant and iXase inhibitory activities of SnFG and its derived oligosaccharides were also evaluated.

## 2. Results and Discussion

### 2.1. Extraction and Purification of Polysaccharides

**Figure 2:**
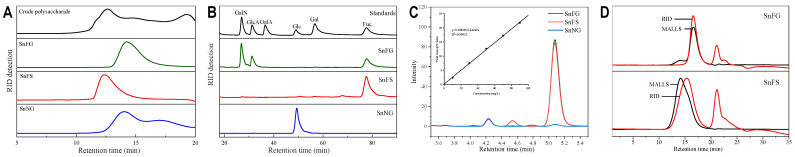
The physicochemical properties of native polysaccharides SnFG, SnFS, and SnNG. (**A**) HPGPC profiles; (**B**) monosaccharide analysis; (**C**) sulfate elution profiles recorded via ion chromatography; (**D**) MALLS-RID chromatograms of SnFG and SnFS.

The crude polysaccharide was extracted from the body walls of *S. naso* by enzymolysis, alkali hydrolysis, and isoelectric precipitation for protein removal. Primary purification was performed via alcohol precipitation for the removal of residual protein and nucleic acid. Then, from this, three polysaccharides were obtained using anion exchange resin in the elution fractions of H_2_O and 0.5 M and 2 M NaCl solutions, designated as SnFS, SnNG and SnFG, with the yield of 2.95%, 0.09% and 0.4%, respectively, based on the dry weight of the body wall. The HPGPC profiles showed that SnFS and SnFG had a good homogeneity, whereas SnNG showed two peaks of different retention times, suggesting a difference in molecular mass (Figure 2A).

### 2.2. Analysis of Physicochemical Properties

Precolumn derivatization with PMP is one of the most effective methods for analyzing the monosaccharide composition of natural polysaccharides. As shown in Figure 2B, the chromatographic profiles demonstrated the presence of only Fuc and Glc in SnFS and SnNG, respectively, whereas three monosaccharides, GalN, GlcA, and Fuc, constituted SnFG. According to the results of ion chromatography profiles (Figure 2C), the OSO_3_^–^ content of SnFS, SnFG and SnNG was calculated to be 31.4%, 34.5%, and 0.6%, respectively. The combined results showed that SnFS was fucan sulfate, and SnNG was a neutral glucan that contained two fractions markedly differing in molecular mass; this was also found in sea cucumber *Pattalus mollis* [37]. SnFG is a fucosylated glycosaminoglycan ubiquitously found in sea cucumbers [9].

As determined by HPGPC-MALLS (Figure 2D), the weight-average molecular mass (Mw) and number-average molecular mass (Mn) of SnFS and SnFG were 411.3 and 304.9 kDa and 61.1 and 58.6 kDa, respectively, as shown in Table 1. The Mw of SnFS was significantly higher than that of SnFG, which is similar to the results found from *S. horrens* and *S. herrmanni* [26,38].

The specific optical rotation of SnFS and SnFG was measured to be –163 and –73, compatible with the residues of L-Fuc. That of SnNG was measured to be +139 (Table 1), revealing the dextrorotatory isomer of D-Glc. These values agreed with those of the corresponding polysaccharides reported in other sea cucumbers [37].

### 2.3. Spectral Analysis of the Natural Polysaccharides

**Figure 3:**
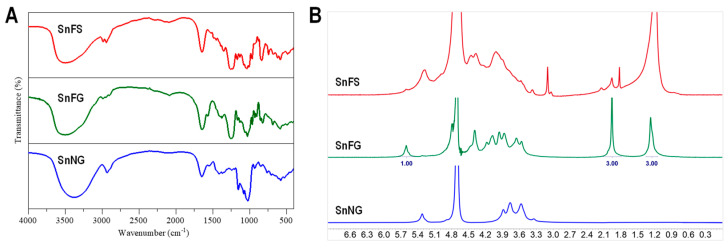
The spectra of SnFG, SnFS, and SnNG. (**A**) FT−IR spectra; (**B**) ^1^H NMR spectra.

FT-IR spectroscopy is a well-established technique to obtain information about functional groups and the structural characteristics of polysaccharides by typically targeting the mid-IR region. In the spectra of SnFS, SnFG and SnNG (Figure 3A), the strong and broad absorption at 3600–3200 cm^−1^ was attributed to the stretching vibration of the hydroxyl group, evident in the spectra of polysaccharides. The absorption peak at 2950–2900 cm^−1^ was assigned as the stretching vibration of C-H. The bands at about 1650 cm^−1^ in SnFS and SnNG were from crystalline water, but the strong peak in SnFG was superimposed on the stretching vibrations of the C=O of acetyl and carboxyl groups. The weak absorption at 1570 cm^−1^ in SnFG should be accountable to the N-H vibrations of the N-acetyl group (GalNAc). Moreover, the obvious absorption at 1260–1250 cm^−1^ in SnFS and SnFG corresponded to the S=O asymmetric stretching vibration of the sulfate group. These characteristic functional groups obtained were in good agreement with the previous results of physicochemical property analyses [37,39].

The ^1^H NMR spectra of SnFS, SnFG and SnNG are displayed in Figure 3B.

For SnFS, the methyl protons of Fuc resonated at ~1.2 ppm, and one anomeric proton was observed in the downfield region at 5.0–5.7 ppm. This suggests that SnFS has a simple structure with only one sulfate pattern of Fuc. The residual proton signals at 3.5–4.7 ppm overlapped heavily as broad peaks.

In the spectrum of SnFG, the methyl protons of Fuc (-CH_3_) and GalNAc (-COCH_3_) resonated at ~1.3 and ~2.0 ppm with an approximately integral area. Only one set of anomeric protons of the α anomer at ~5.6 ppm hinted that SnFG was mostly branched by Fuc_2S4S_, according to previous studies [26,38].

For SnNG, its signals seemed clear and simple, clustering in the region of 3.3–3.9 ppm, because it consists of neutral Glc residues, while the resonances at 5.3 ppm indicated an α anomer of Glc. A neutral glucan was previously reported from sea cucumbers *Holothuria edulis* and *P. mollis* and was presumed to structurally resemble animal glycogen [37,39].

### 2.4. Hydrogen Peroxide Depolymerization of SnFS and Its Structural Elucidation

**Figure 4:**
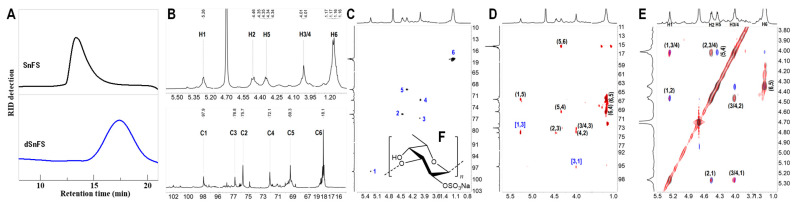
(**A**) HPGPC profiles of the native SnFS and dSnFS; (**B**) ^1^H and ^13^C NMR spectra and chemical shift assignments of dSnFS; (**C**) HSQC spectrum and the signal assignments of dSnFS; (**D**) HMBC spectrum and the signal assignments of dSnFS; (**E**) the superimposed ^1^H-^1^H NMR spectra (COSY—gray, TOCSY—red, ROESY—blue) of dSnFS; (**F**) the structure of dSnFS.

Due to the high molecular weight and the severe overlapping of proton signals of SnFS, depolymerization was carried out using H_2_O_2_ to clarify its structure. As shown in Figure 4A, the retention time of dSnFS was significantly delayed and the molecular weight was markedly reduced compared with the prototype SnFS.

Compared with the ^1^H NMR spectrum of SnFS, the agreements of the signals and chemical shifts of dSnFS indicated that the structural features remain stable after depolymerization (Figure 3B and Figure 4B). The six correlations in the ^1^H-^13^C HSQC spectrum of dSnFS infer that SnFS only consists of one sulfation pattern of Fuc residues. As assigned in Figure 4C–E, the chemical shifts of H2 and C2 at 4.46 and 75.7 ppm indicate that sulfate substitution occurred at the O-2 position of Fuc. Those of H3 and C3 at 4.02 and 76.8 ppm reveals the glycosylation at the O-3 position. This linkage was also confirmed from the cross peaks of H1/C3 and H3/C1 in the ^1^H-^13^C HMBC spectrum. Thus, dSnFS, as well as SnFS, was deduced to have a highly regular structure: {3)-L-Fuc_2S_-(α1,}_n_ (Figure 4F).

### 2.5. β-Eliminative Depolymerization of SnFG and the Purification of Oligosaccharides

Selective depolymerization of natural FG, in combination with the analyses of its derived oligosaccharides, is one of the most powerful strategies for deciphering its exact structure. Chemical β-elimination could selectively break the D-GalNAc-(β1→4)-D-GlcA glycosidic bonds, thus forming Δ^4,5^ unsaturated glucuronic acid at the non-reducing end of the products, resulting in a maximum UV absorption at 232 nm [40]. Using this method, the complex FG from sea cucumbers *Ludwigothurea grisea*, *Acaudina molpadioide*, *Holothuria nobili*, and *Phyllophorella kohkutiensis* was unambiguously identified in recent studies [7,8,9,10]. These structurally well-defined oligosaccharides could be used for further structure–activity relationship analyses.

**Figure 5:**
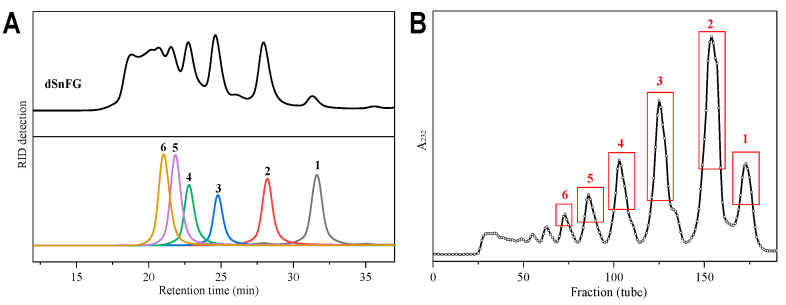
(**A**), HPGPC profiles of dSnFG and oligosaccharides **1**–**6** (Superdex peptide 10/300 GL column); (**B**) elution curve of dSnFG using the Bio-Gel P10 column and monitored via UV absorption at 232 nm.

Herein, β-eliminative depolymerization was performed on SnFG to obtain its low-molecular-weight product dSnFG, with a yield of 60%. The HPGPC profile of dSnFG suggested that it consisted of a series of oligosaccharides of different molecular weights (Figure 5A). dSnFG was therefore fractionated and purified by Bio-gel P-10. The elution curve presented in Figure 5B shows the effective isolation of its oligomers. Finally, six purified oligosaccharides (**1**–**6**) were obtained for structural analysis.

### 2.6. Structural Analyses of Oligosaccharides **1**–**6** and SnFG

**Figure 6:**
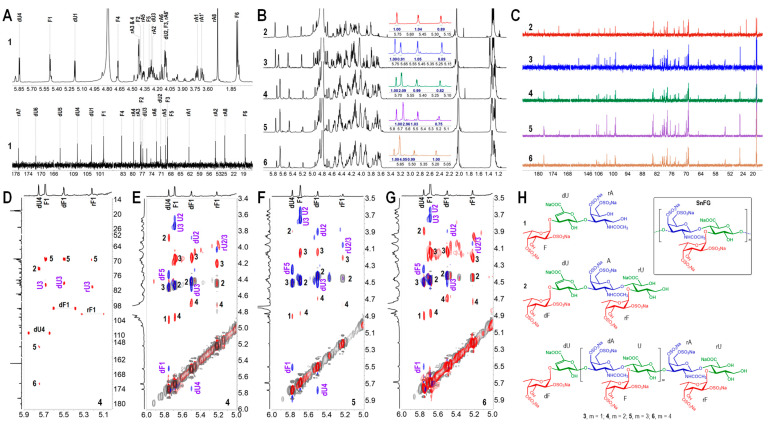
(**A**) ^1^H and ^13^C NMR spectra and chemical shift assignments of **1**; (**B**) ^1^H NMR spectra of **2**–**6**; (**C**) ^13^C NMR spectra of **2**–**6**; (**D**) partial HMBC spectrum and the signal assignments of **4**; (**E**) the superimposed ^1^H-^1^H NMR spectra (COSY—gray, TOCSY—red, ROESY—blue) and partial signal assignments of **4**–**6**: (**E**) **4**, (**F**) **5**, (**G**) **6**; (**H**) the structure of **1**–**6** and the supposed structure of SnFG. Some NMR spectra were processed appropriately on MestReNova software, with the removal of the non-signal region to amplify the signal region.

The ^1^H NMR and ^13^C NMR spectra of **1** were similar to that of the trisaccharide L-Fuc_2S4S_-(α1,3)-L-Δ^4,5^HexA-(α1,3)-D-GalNAc_4S6S_-*ol* we reported before [38]. The ^1^H and ^13^C chemical shifts were assigned as in Figure 6A. In particular, the downfield signals of H/C-2 (4.40, 77.4 ppm) and H/C-4 (4.67, 83.5 ppm) confirmed the sulfation at O-2 and O-4 of Fuc. Trisaccharides are easily accessible from the β-eliminative depolymerization product, as it is the simplest structural unit of FG.

With **2**–**6**, as seen in the stacked ^1^H NMR spectra (Figure 6B), the chemical shifts of the proton signals appeared at similar locations and showed regular variations, especially at the region of 5.2–5.8 ppm, where the signals are clear and easy to discern. With **2**, the integral of the three protons at 5.2, 5.5 and 5.77 ppm was approximate. Among them, 5.77 ppm was attributed to the H-4 of Δ^4,5^ unsaturated glucuronic acid (dU), derived from the breaking of glycosidic bonds in *D*-GalNAc-β(1,4)-*D*-GlcA. In its ^13^C NMR spectrum (Figure 6C and Figure S1), four resonances in the anomeric region of 98–107 ppm suggested that **2** was a heptasaccharide, as the hemiacetal at the reducing end was reduced to the sugar alcohol using NaBH_4_ (Figures S2 and S3). One more alpha proton signal at 5.2–5.8 ppm was observed in the ^1^H NMR spectrum of **3** than **2**. The seven carbon resonances at 100–109 ppm implied **3** to be an octasaccharide (Figures S4–S6). The two structures remind us of the hepta- and octasaccharides derived from the depolymerized FG from sea cucumber *S. horrens* we identified before [38]. In comparison, **2** was a heptasaccharide with the structure of *L*-Fuc_2S4S_-α(1,3)-L-Δ^4,5^HexA-α(1,3)-*D*-GalNAc_4S6S_-β(1,4)-[*L*-Fuc_2S4S_-α(1,3)]-*D*-GlcA-*ol*, and **3** was an octasaccharide with the structure of *L*-Fuc_2S4S_-α(1,3)-*L*-Δ^4,5^HexA-α(1,3)-*D*-GalNAc_4S6S_-β(1,4)-[*L*-Fuc_2S4S_-α(1,3)]-*D*-GlcA-β(1,3)-*D*-GalNAc_4S6S_-β(1,4)-[*L*-Fuc_2S4S_-α(1,3)]-*D*-GlcA-*ol*, with one more trisaccharide unit than **2**. All the ^1^H and ^13^C signals are assigned in Table 2. The proton signals at 5.22 and 5.50 ppm were from the H-1 of α-*L*-Fuc_2S4S_ linked to glucuronic acid residues (rU and dU), as the signals at O-2 and O-4 clearly shifted downfield (4.4/78 ppm; 4.7/84 ppm). The signal at 5.69 ppm in the ^1^H NMR spectrum of **3** was assigned to the H-1 of α-*L*-Fuc_2S4S_ (F1) that is linked to U3. Fucosylation at the O-3 of (r/d)U could be deduced from the chemical shifts of U3 (δ*_C_* ~80 ppm). In addition, all the GalNAc residues r/dA were sulfated at O-4 and O-6 sites.

For the structure of **4**, it is easy to observe that the four proton signals appearing at 5.0–5.7 ppm remained essentially the same as in **3**, whereas the integral area at 5.67 ppm increased, suggesting that there may be one more Fuc; thus, it contains one more trisaccharide unit than **3**. In the HMBC spectrum of **4** (Figure 6D and Figure S5), the correlations of rF1/rU3, dF1/dU3, and F1/U3 marked in purple revealed that all the Fuc residues were connected to O-3 at GlcA residues. The linkages were confirmed by the correlations marked in purple in the superimposed ^1^H-^1^H COSY/TOCSY/ROESY spectra (Figure 6E and Figure S6). From the spectra, 2,4-di-O-sulfation of all the Fuc molecules was also proven. Thus, **4** was proved to be a hendecasaccharide.

Likewise, **5** is a tetradecasaccharide consisting of one more trisaccharide unit than **4**. The linkage positions and sulfation patterns of Fuc branches were confirmed from the ^1^H-^1^H correlations in Figure 6F, Figures S7 and S8. **6** was proven to be a heptadecasaccharide (Figure 6G). The ESI-Q-TOF MS (Figure S9) assignments of the oligosaccharides **2**–**6** are listed in Table 3. All the structures of oligosaccharides are presented in Figure 6H.

Comparison of the structures of the oligosaccharides **1**–**6** showed that the structure regularly lengthened with an increase in the degree of polymerization (dp). The oligosaccharides **2**–**6** incrementally contained an additional trisaccharide structural unit one by one, except **1, 2**–**6** underwent a peeling reaction to lose a sugar group at the reducing end under such alkaline conditions. According to the structural features, it could be seen that all the GalNAc residues were 2,4-di-O-sulfated, forming its backbone, like chondroitin sulfate E. All the branches were Fuc_2S4S_ attached to the O-3 of GlcA. Consequently, SnFG possessed the structure of {D-GalNAc_4S6S_-β(1,4)-[L-Fuc_2S4S_-α(1,3)]-D-GlcA}, determined using the bottom-up strategy (Figure 6H).

### 2.7. Anticoagulation and Inhibition of iXase of Oligosaccharides **3**–**6** and SnFG

Previous studies have demonstrated that natural FG potently affects the intrinsic coagulation pathway by targeting the Xase complex (iXase). Simultaneously, it could affect the common coagulation pathway to a certain extent and inhibit IIa inhibition in the presence of antithrombin [14,21,22]. Depolymerized FG with a certain molecular weight could potently and selectively inhibit iXase, whereas no obvious effects on the extrinsic and common coagulation pathways were detected. Octasaccharide was the smallest size molecule capable of this potent inhibition [28,29].

**Figure 7:**
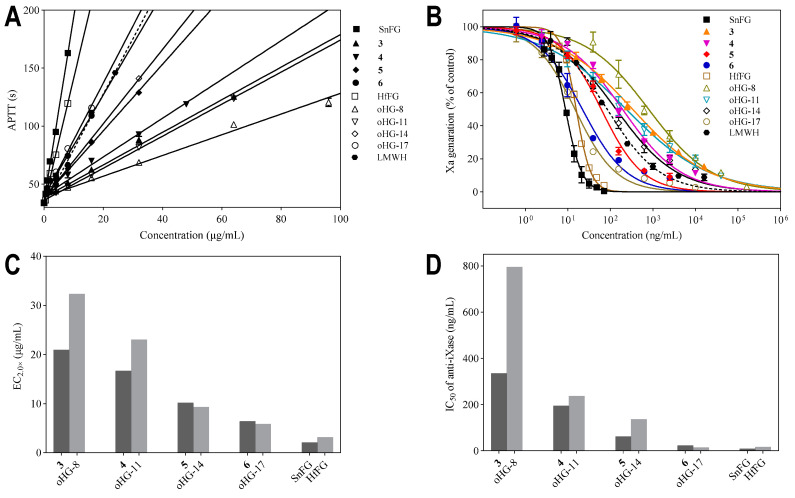
The effects of SnFG, HfFG, and the derived oligosaccharides on APTT (**A**) and iXase (**B**); EC_2_._0×_ (**C**) and IC_50_ values (**D**) of oligosaccharides with the same degree of polymerization.

In this work, we obtained for the first time a series of Fuc_2S4S_-containing oligosaccharides with a degree of polymerization (dp) up to 17, taking advantage of the simple structure of SnFG. This allowed us to explore the effect of the dp on the activity of oligosaccharides only containing Fuc_2S4S_ branches. Herein, the effects of natural SnFG and its derived oligosaccharides **3**–**6** on the intrinsic coagulation pathway and the inhibition of iXase were evaluated. For comparative analyses, the activities of the Fuc_3S4S_-branching FG oligosaccharides from the sea cucumber *H. fuscopunctata* (oHG-8, oHG-11, oHG-14, and oHG-17) were also assayed in parallel (Figure 7).

As shown in Table 4 and Figure 7, SnFG inhibited the intrinsic pathway at a concentration of 2.07 μg/mL required for double APTT, slightly stronger than HfFG (3.19 μg/mL). In the comparison of the two series of oligosaccharides, **3** was about 1.5-fold stronger than oHG-8 (EC_2_._0×_, 20.94 vs. 32.35 μg/mL), confirming that Fuc_2S4S_ branches contributed more than Fuc_3S4S_ branches in octasaccharides, which agrees with our previous reports. The potency increased with the increase in the dp. However, with the same dp, the gap in the inhibition potency gradually decreased between the oligosaccharides containing Fuc_2S4S_ and Fuc_3S4S_ branches [29]. The concentrations of the two heptadecasaccharides (**6** and oHG-17) required to double the APTT were almost equal (EC_2_._0×_, 6.41 vs. 5.87 μg/mL).

Effects on the iXase complex showed that SnFG and HfFG potently inhibited iXase activity with the IC_50_ values of 8.80 and 15.74 ng/mL, nearly 10-fold stronger than the positive control LMWH. From the results of the structure–activity relationship analysis, **3,** branched by Fuc_2S4S_, inhibited iXase more potently than oHG-8 that is branched by Fuc_3S4S_. Similar to the trend in the effects on the intrinsic coagulation pathway, the inhibitory activity of oligosaccharides on iXase increased with the increase in the dp. oHG-17 showed the approximate activity with its prototype HfFG. In the previous study, we observed that the inhibition potency remained essentially stable until the dp reached 17 [30], whereas the heptadecasaccharide branched by Fuc_2S4S_ (**6**) did not reach the strength of its prototype SnFG.

In short, the effect of the sulfate branch pattern on anticoagulation and iXase was more pronounced in the relatively low-dp oligosaccharides. As the dp increased, the effect of sulfation was swamped by the effect of the chain length.

## 3. Materials and Methods

### 3.1. Materials and Reagents

The dried body wall of sea cucumber *S. naso* was purchased from Zhanjiang city, Guangdong province, China. Species identification was completed based on the partial 16S ribosomal RNA gene with a similarity of 99.34%. The amberlite FPA98 anion-exchange resin was from Rohm and Haas Company (Louisiana, USA). Sephadex G25 and Bio-Gel P10 were from GE Healthcare company and Bio-Rad Laboratories, respectively. The Agilent 1260 high-performance liquid chromatograph was from Agilent (California, USA). The standard monosaccharides N-acetyl-D-galactosamine (GalNAc), D-glucuronic acid (GlcA), D-galacturonic acid (GalA), D-galactose (Gal), D-glucose (Glc) and L-fucose (Fuc) were purchased from Sigma-Aldrich (Shanghai, China) or Aladdin Reagents LTD (Shanghai, China). Benzyl chloride was purchased from Aladdin Chemistry Co. Ltd (Shanghai, China). Deuterium oxide (99.9% atom D) was obtained from Sigma-Aldrich (Shanghai, China). The activated partial thromboplastin time (APTT) reagent and human coagulation control plasma were from Teco Medical (Berlin, Germany). Biophen FVIII: C kits and Biophen Anti-Xa kits were from HYPHEN BioMed (Neuville-sur-Oise, France). Human factor VIII was from Bayer HealthCare LLC (Leverkusen, Germany). All other chemicals were of reagent grade and obtained commercially.

### 3.2. Purification of the Polysaccharides

The extraction procedure of crude polysaccharide from *S. naso* followed a previous description [9]. A total of 185 g of dried species was crushed, and then 1.85 L of water and 1.8 g of papain were added and reacted at 50 °C for 6 hours (h). After the reaction, the temperature was raised to 90 °C for 20 min, followed by treatment with alkali using a final concentration of 0.25 M sodium hydroxide at 45 °C for 2 h. The extraction solution was cooled to room temperature, the pH was adjusted to ~2 with 6 M HCl, and the solution refrigerated overnight and centrifuged to remove the precipitate. The supernatant was neutralized, followed by addition of 3.5 L ethanol (95%), and then centrifuged (4000 rpm, 15 min) to obtain a precipitate. After re-dissolution, another graded alcohol precipitation (60%, *v/v*) was carried out with the addition of 0.5 M potassium acetate to yield the precipitate as the crude polysaccharide.

The crude polysaccharide underwent purification using strong anion exchange column chromatography FPA98 (Cl^−^), with the elution of water and 0.5 M, 1.0 M, 1.5 M, 2.0 M, and 3.0 M NaCl solutions successively. Each fraction was analyzed for the presence of samples using an Agilent 1260 HPLC system equipped with a Shodex OH-pak SB-804 HQ column under the following conditions: column temperature, 35 °C; mobile phase, 0.1 M NaCl; flow rate, 0.5 mL/min; and a differential refractive detector (RID). Samples were subsequently desalted and lyophilized.

### 3.3. Analysis of Physicochemical Properties

Analysis of monosaccharide composition was performed using a precolumn derivatization method with 1-phenyl-3-methyl-5-pyrazolone (PMP) [9]. Each polysaccharide was hydrolyzed to a monosaccharide using trifluoroacetic acid (TFA, 2M) at 110 °C for 4 h, and the monosaccharides were derivatized with PMP at 70 °C for 0.5 h under alkaline conditions. The products were analyzed using an Agilent 1260 HPLC system and the chromatographic conditions were as follows: ZORBAX Eclipse Plus C18 (4.6 × 250 mm) column, column temperature: 30 °C, injection volume: 10 μL, mobile phase: 20 mM ammonium acetate buffer (pH 5.5): acetonitrile = 83:17, flow rate: 1.0 mL/min, a diode array detector (DAD), and wavenumber: 250 nm.

The specific optical rotation was determined via a Autopol IV Automatic Polarimeter (Rudoiph). Data for each polysaccharide were collected three times at 25 °C to take the average value.

The molecular mass was measured via multi-angle laser scattering (HPGPC-MALLS) using Agilent technologies 1260 series high-performance liquid chromatograms equipped with a Shodex OHpak SB-806 HQ (8 mm × 300 mm) column. The detectors were a RID and a MALLS (DAWN HELEOS-I). The refractive index increase (*dn/dc*) was 0.119 mL/g.

The sulfate content of each sample was determined via ion chromatography (IC) using a Dionex ICS2100 ion chromatography system (Thermo Fisher Scientific, USA) [10,41]. A series of sulfate standard solutions (10, 30, 50, 70, and 90 mg/L) was recorded to establish the standard curve. Each sample was first hydrolyzed with TFA at 110 °C for 8 h and the hydrolysate was quantified after removing the excess TFA. The retention time and peak area of sulfate were recorded via IC, and then the sulfate content of each polysaccharide was calculated according to the standard curve.

### 3.4. IR, NMR and MS Spectral Analyses

Fourier transform infrared spectroscopy (FT-IR) of SnFG, SnFS and SnNG was performed in KBr pellets on an IRTracer-100 infrared spectrometer in the range of 4000–400 cm^−1^.

The ^1^H NMR spectra of SnFG, SnFS, SnNG, and dSnFS were obtained with a Bruker Advance III 600 MHz spectrometer at 298 K. The 1D/2D NMR spectra of dSnFS and the oligosaccharides from dSnFG were recorded on an Advance III 800 MHz NMR spectrometer at 298 K.

ESI-Q-TOF-MS of the oligosaccharides was conducted on a 6540 UHD accurate-mass Q-TOF LC/MS spectrometer.

### 3.5. Chemical Depolymerization of Polysaccharides

#### 3.5.1. Hydrogen Peroxide Depolymerization of SnFS

A total of 100 mg of SnFS was dissolved in 3.65 mL of deionized water, followed by the addition of 0.3 mg copper acetate and 0.45 mL 30% H_2_O_2_. The reaction mixture was stirred at 35 °C for 3 h, and the depolymerization product dSnFS was obtained after alcohol precipitation and desalinization.

#### 3.5.2. β-Eliminative Depolymerization of SnFG

A total of 200 mg of SnFG was dissolved in 3 mL of deionized water, then mixed with a benzethonium chloride (500 mg) solution to transform SnFG to its solid quaternary ammonium salt, which was dried under vacuum to a constant weight (552 mg). The solid was dissolved in N,N-dimethylformamide (2.76 mL) and reacted with benzyl chloride for 24 h at 35 °C. Then, freshly prepared EtONa/EtOH (0.16 M) was added for a duration of 30 min at 25 °C for depolymerization. Following this step, a saturated sodium chloride solution was added to re-exchange the depolymerized SnFG (dSnFG) to the sodium salt form. dSnFG was then reduced using NaBH_4_, and the residual benzyl group on the carboxyl group was removed by NaOH. After neutralization, desalinization and lyophilization, dSnFG was collected as a white solid.

### 3.6. Purification of Oligosaccharides from dSnFG

dSnFG was loaded onto a Bio-gel P-10 column, and eluted with 0.2 M NaCl at a flow rate of 6.6 mL/h. A total of 2 mL of eluent was collected per tube and monitored with a UV-Vis spectrophotometer at 232 nm. The elution curve was plotted based on A_232_, and selected samples were analyzed via HPGPC (Superdex Peptide 10/300 GL column, 10 × 300 mm). The oligosaccharides with the same molecular mass were combined according to the elution curve and results of HPGPC profiles, and were finally obtained after desalinization and lyophilization.

### 3.7. Anticoagulant Activity and Inhibition of iXase of SnFG and Its Derived Oligosaccharides

The anticoagulant activities of natural SnFG and its oligosaccharides **3**, **4**, **5**, and **6** were assessed using a coagulometer (TECO, MC-2000) using APTT reagents and standard human plasma, according to a previous method [10]. Briefly, 5 μL of a series of concentrations of the samples was added to a standard preheated to 37 °C and another 45 μL of standard human plasma was added for co-incubation at 37 °C for 2 min, followed by the addition of 50 μL of APTT reagent for incubation for another 3 min. Subsequently, 50 μL of CaCl_2_ (0.02 M) was added and the timer was started.

Inhibition of iXase was assessed using the chromogenic substrate assay and the FVIII assay kit BIOPHEN FVIII:C: containing R1 (FX), R2 (Activation Reagent), and R3 (SXa-11) and recombinant human factor VIII (FVIII) [30]. A total of 30 μL of each sample was added to the 96-well plate, followed by 30 μL of R2 and 30 μL of FVIII solution (2 IU/mL), and the place was placed in the microplate reader. The plates were mixed and incubated at 37 °C for 2 min. Then, 30 μL of R1 was added to mix and incubated at 37 °C for 1 min, and finally, 30 μL of R3 was added. The absorbance value at 405 nm (OD_405nm_) was recorded via a microplate reader, and continuous reading was performed for 2min at 15 s intervals. The experiments were performed in duplicate. The average value of OD_405nm_ was linearly fitted to time, and the slope (the rate of change in absorbance value ΔOD_405nm_/min) corresponded to the iXase activity.

Activities of natural HfFG (FG from *H. fuscopunctata*) [42] and its oligosaccharides oHG-8, oHG-11, oHG-14, and oHG-17 were simultaneously detected for comparison [30].

## 4. Conclusions

FS and FG are two common acidic polysaccharides rich in fucose that are found in the body walls of sea cucumbers. However, they exhibit significant structural differences. Generally, FS is composed solely of fucose and sulfate groups, forming a linear polysaccharide. The patterns of sulfation and the glycosidic linkages vary depending on the species of the sea cucumber, thus generating various FSs with different repetitive structural units. FG is composed of a CS-E backbone branched with sulfated fucose. A recent investigation showed that fucose undergoes glycosylation with Gal or GalNAc.

*Stichopus naso*, belonging to the genus *Stichopus*, is a low-value sea cucumber available on the market, and there have been no reports on the structure of its polysaccharides. In this study, crude polysaccharides were obtained from the body wall of *S. naso* through enzymatic hydrolysis and alkaline treatment, followed by ethanol precipitation. Three polysaccharides, SnFS, SnNG, and SnFG, were purified using strong anion exchange chromatography. The physicochemical properties were analyzed from the aspects of homogeneity, sulfate content, monosaccharide composition, specific optical rotation, and infrared absorbance.

SnNG contains two neutral glucans with different molecular weights, possessing structures like glycogen. SnFS has a higher molecular weight compared to common FS. Low-molecular-weight products, dSnFS, were prepared through oxidative degradation. A detailed NMR structural analysis was conducted to confirm that SnFS has a highly regular structure of {3)-L-Fuc_2S_-(α1,}_n_. With SnFG, selective β-elimination degradation was employed and from the depolymerized products, tri-, penta-, octa-, hendeca-, tetradeca-, and heptadecasaccharides were purified and clarified. The backbone all derived CS-E-like disaccharide repetitive units and branches was of the Fuc_2S4S_ type. Based on the structural characteristics of the series of oligosaccharides, it was deduced that natural SnFG possesses a regular trisaccharide repeating structural unit {*D*-GalNAc_4S6S_-β(1,4)-[*L*-Fuc_2S4S_-α(1,3)]-*D*-GlcA} using the bottom-up strategy. For the first time, purified oligosaccharides (branched by Fuc_2S4S_) with a dp up to 17 were obtained, which facilitates the differentiation of the effects of molecular weight and sulfate content on the anticoagulation activity and the structure–activity relationships of this series of oligosaccharides. Through a comparative analysis with the series of oligosaccharides solely branched with Fuc_3S4S_, it could be found that in oligosaccharides with a lower dp, such as octasaccharides, Fuc_2S4S_ prolongs the APTT and increases iXase inhibition; as the dp increases, the influence from the sulfation pattern diminishes, until it is overshadowed by the effects of the molecular weight. The discovery that SnFS and SnFG are both structurally the simplest version of natural FG and FS could facilitate the application and development of low-value *S. naso* sea cucumbers.

## Figures and Tables

**Table 1:** Chemical compositions and physicochemical properties of SnFG, SnFS, and SnNG.

	Monosaccharide Composition				[α]	Mw	Mn	OSO_3_^−^ (%)
	GlcA	GalN	Fuc	Glc		(kDa)		
SnFS	˗	˗	+	˗	−163	411.3	304.9	31.4
SnFG	+	+	+	˗	−73	61.1	58.6	34.5
SnNG	˗	˗	˗	+	+139	/	/	0.6

**Table 2:** ^1^H/^13^C NMR chemical shift assignments of oligosaccharides **2** and **3** (δ, ppm; 600 MHz, D_2_O).

Comp.		dF	rF	dU	rU	A	F	U	dA	rA
**2**	H1	5.47	5.20	4.88	3.77	4.76				
	C1	99.1	101.3	105.8	65.0	103.4				
	H2	*4.38 ^a^*	*4.40*	3.85	4.01	4.07				
	C2	*77.7*	*78.0*	73.0	73.3	54.3				
	H3	4.10	4.18	**4.46** ^b^	**4.01**	4.19				
	C3	69.0	69.1	**79.0**	**80.7**	78.5				
	H4	*4.66*	*4.70*	5.73	4.13	*4.96*				
	C4	*83.4*	*83.7*	109.1	82.9	*79.1*				
	H5	4.32	4.55		4.28	4.08				
	C5	69.0	69.4	149.6	74.9	75.0				
	H6/6’	1.27	1.29		180.0	*4.31/4.17*				
	C6	18.4	18.7	171.6		*70.5*				
	C7					177.6				
	H8					2.02				
	C8					25.2				
**3**	H1	5.49	5.22	4.89	3.79		5.69	4.45	4.60	4.76
	C1	99.0	101.3	106.6	65.0		99.2	105.9	102.5	103.4
	H2	*4.41*	*4.43*	3.89	4.04		4.49	3.65	4.13	4.04
	C2	*77.7*	*78.0*	73.0	73.2		77.7	76.5	54.0	54.2
	H3	4.14	4.21	**4.51**	**4.04**		4.14	**3.74**	4.15	4.04
	C3	69.2	69.1	**79.1**	**80.6**		69.2	**79.9**	78.7	78.8
	H4	*4.69*	*4.72*	5.75	4.18		4.86	3.95	*4.97*	*4.82*
	C4	*83.4*	*83.7*	109.0	83.0		83.9	78.2	*79.0*	*79.0*
	H5	4.35	4.56		4.31		4.93	3.68	4.05	4.05
	C5	69.0	69.4	149.7	74.8		69.0	79.7	74.9	74.5
	H6/6’	1.30	1.32				1.38		*4.28/4.20*	*4.30/4.20*
	C6	18.4	18.6	171.6	180.0		18.5	177.7	*70.0*	*70.5*
	C7								177.7	177.7
	H8								2.05	2.05
	C8								25.2	25.2

^a^ Values in italics indicate positions of sulfation; ^b^ values in bold indicate the branch sites of Fuc.

**Table 3:** Negative-ion ESI-MS of oligosaccharides **2**–**6**.

Comp.	Molecular Ion	*m/z*		Molecular Formula	Mw
		Observed	Calculated		
**2**	[M-2Na]^2−^	729.4473	729.4465	C_32_H_43_NNa_6_O_43_S_6_^2−^	1505.9382
	[M-4Na + 2H]^2−^	707.4650	707.4645	C_32_H_45_NNa_4_O_43_S_6_^2−^	
	[M-6Na + 4H]^2−^	685.4821	685.4826	C_32_H_47_NNa_2_O_43_S_6_^2−^	
	[M-5Na + 2H]^3−^	463.9813	463.9800	C_32_H_45_NNa_3_O_43_S_6_^3−^	
**3**	[M-5Na + 2H]^3−^	782.2808	782.2821	C_52_H_71_N_2_Na_8_O_70_S_10_^3−^	2461.5350
	[M-6Na + 3H]^3−^	774.9552	774.9548	C_52_H_72_N_2_Na_7_O_70_S_10_^3−^	
	[M-7Na + 4H]^3−^	767.6271	767.6274	C_52_H_73_N_2_Na_6_O_70_S_10_^3−^	
	[M-8Na + 4H]^4−^	569.9748	569.9733	C_52_H_73_N_2_Na_5_O_70_S_10_^4−^	
**4**	[M-9Na + 5H]^4−^	803.7056	803.2044	C_72_H_100_N_3_Na_9_O_97_S_14_^4−^	3414.6842
	[M-8Na-SO_3_Na + 5H]^4−^	783.2174	783.2152	C_72_H_100_N_3_Na_9_O_94_S_13_^4−^	
	[M-9Na + 4H]^5−^	642.7641	642.3620	C_72_H_99_N_3_Na_9_O_97_S_14_^5−^	
	[M-11Na + 6H]^5−^	633.5697	633.5693	C_72_H_101_N_3_Na_7_O_97_S_14_^5−^	
**5**	[M-6Na]^6−^	705.8212	705.2759	C_92_H_121_N_4_Na_17_O_124_S_18_^6−^	4369.5905
	[M-7Na]^7−^	601.0156	601.2380	C_92_H_121_N_4_Na_16_O_124_S_18_^7−^	
**6**	[M-11Na + 4H]^7−^	725.8486	725.2354	C_112_H_150_N_5_Na_21_O_152_S_22_^6−^	5325.5002
	[M-7Na + H + H_2_O]^6−^	863.1834	863.9322	C_112_H_150_N_5_Na_21_O_152_S_22_^6−^	
	[M-4Na-3SO_3_Na + H + 2H_2_O]^6−^	826.7962	826.9556	C_112_H_152_N_5_Na_21_O_144_S_19_^6−^	

**Table 4:** Anticoagulant activities and iXase inhibition of SnFG, HfFG, and their oligosaccharides.

Sample	Resource	Branch Type	dp	Mw ^a^ (Da)	2APTT ^c^ (μg/mL)	Anti-iXase ^d^ (ng/mL)
**3**	*Stichopus naso*	Fuc_2S4S_	8	2462	20.94 ± 0.27	335.0 ± 24.73
**4**			11	3417	16.68 ± 0.29	194.1 ± 18.81
**5**			14	4373	10.19 ± 0.05	61.14 ± 5.37
**6**			17	5328	6.41 ± 0.16	21.99 ± 3.04
SnFG				61,100 ^b^	2.07 ± 0.02	8.80 ± 0.28
oHG-8	*Holothuria fuscopunctata*	Fuc_3S4S_	8	2462	32.35 ± 0.30	795.4 ± 98.84
oHG-11			11	3417	23.00 ± 0.06	237.0 ± 30.41
oHG-14			14	4373	9.31 ± 0.05	136.6 ± 20.87
oHG-17			17	5328	5.87 ± 0.08	14.75 ± 1.31
HfFG				42,600 ^b^	3.19 ± 0.02	15.74 ± 0.29
LMWH ^e^				4500	7.46 ± 0.02	84.44 ± 7.99

^a^ Molecular mass was obtained according to its well-defined structure; ^b^ molecular mass was calculated by GPC software; ^c^ concentration required to double the APTT of human plasma. Results are expressed as EC_2_._0×_ ± SD (n = 2); ^d^ concentration required to inhibit 50% Xase activity. Results are expressed as IC_50_ ± SE (n = 2); ^e^ positive control. LMWH: enoxaparin.

## Data Availability

All data presented in this study are available from the corresponding author on reasonable request.

## Supplementary Materials

The following supporting information can be downloaded at https://www.mdpi.com/article/10.3390/md22060265/s1, Figure S1. The full ^13^C NMR spectra of **2** − **6**. Figure S2. The ^1^H and ^13^C NMR spectra and signal assignments of **2**. Figure S3. The HSQC spectrum and signal assignments of **2**. Figure S4. The HSQC spectrum and signal assignments of **3**. Figure S5. The superimposed ^1^H−^13^C HSQC and HMBC spectra and signal assignments of **4**. Figure S6. The superimposed ^1^H−^1^H COSY, TOCSY, ROESY and signal assignments of **4**. Figure S7. The superimposed ^1^H−^13^C HSQC and HMBC spectra and signal assignments of **5**. Figure S8. The superimposed ^1^H−^13^C HSQC and HMBC spectra and signal assignments of **5**. Figure S9. ESI-Q-TOF MS spectra of **2**–**6**.
