# Supplementary material for: Purification and Structural Analyses of Sulfated Polysaccharides from Low-Value Sea Cucumber Stichopus naso and Anticoagulant Activities of Its Oligosaccharides
Source: Mar Drugs. 2024 Jun 8;22(6):265. doi: 10.3390/md22060265 (PMC11204762; doi:10.3390/md22060265)
Supplement: Supplementary file 1 [file marinedrugs-22-00265-s001.zip › marinedrugs-3033250-supplementary.pdf]

Supporting Information

Purification and Structural Analyses of Sulfated Polysaccharides from Low-Value Sea Cucumber *Stichopus naso* and Anticoagulant Activities of Its Oligosaccharides

Lige Cui[1], Huifang Sun[1], Xiaolei Shang[1], Jing Wen[2], Pengfei Li[1], Shengtao Yang[1], Linxia Chen[1], Xiangyang Huang[1], Haoyang Li[1], Ronghua Yin[1*], Jinhua Zhao[1]

[1] School of Pharmaceutical Sciences, South-Central Minzu University, Wuhan 430074, China

[2] School of Biology and Agriculture, Shaoguan University, Shaoguan, 512005, China

[*] Corresponding Author

E-mail address: yinrh77@163.com (R. Y)

Table of contents

Figure S1. The full ^{13}C NMR spectra of **2** – **6**.

Figure S2. The ^{1}H and ^{13}C NMR spectra and signal assignments of **2**.

Figure S3. The HSQC spectrum and signal assignments of **2**.

Figure S4. The HSQC spectrum and signal assignments of **3**.

Figure S5. The superimposed ^{1}H–^{13}C HSQC and HMBC spectra and signal assignments of **4**.

Figure S6. The superimposed ^{1}H–^{1}H COSY, TOCSY, ROESY and signal assignments of **4**.

Figure S7. The superimposed ^{1}H–^{13}C HSQC and HMBC spectra and signal assignments of **5**.

Figure S8. The superimposed ^{1}H–^{13}C HSQC and HMBC spectra and signal assignments of **5**.

Figure S9. ESI-Q-TOF MS spectra of **2** – **6**.

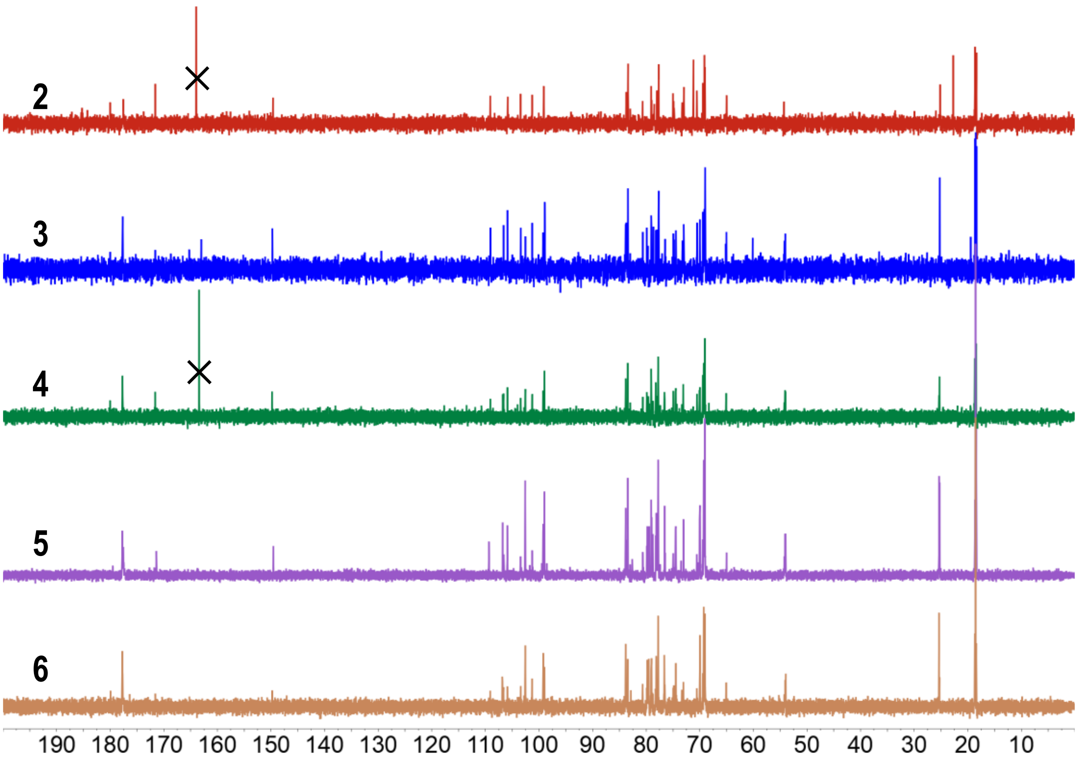

Figure S1. The full ^{13}C NMR spectra of **2** − **6**.

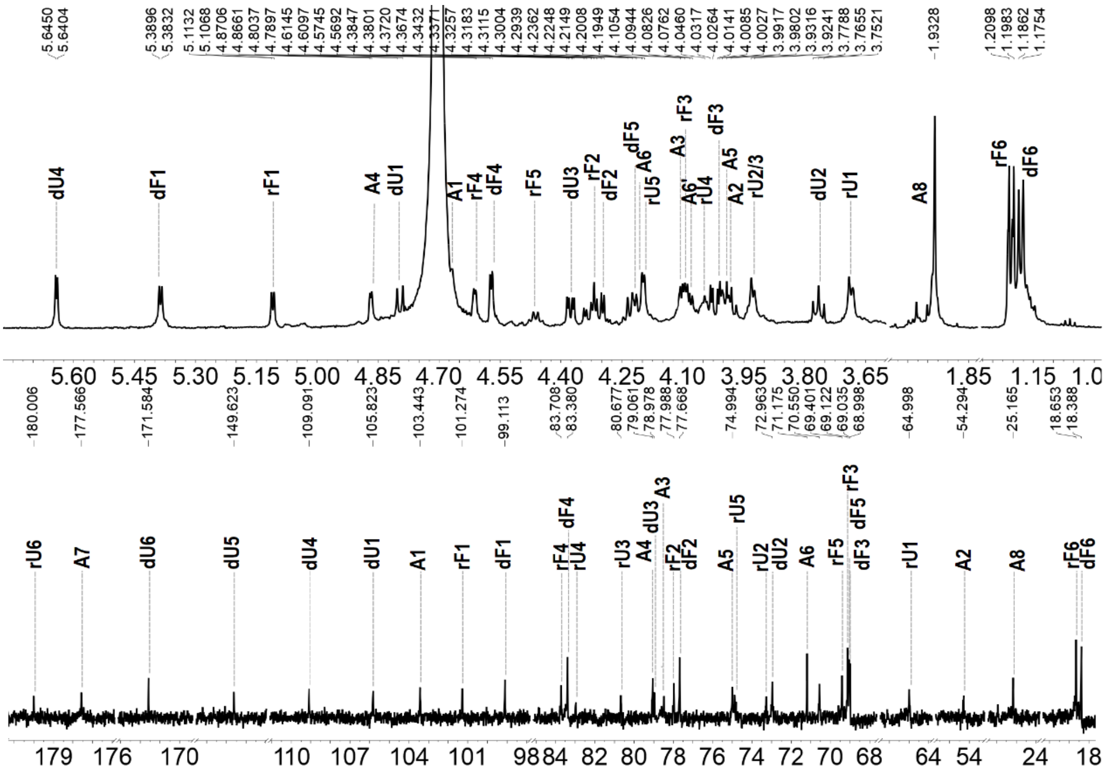

Figure S2. The ^{1}H and ^{13}C NMR spectra and signal assignments of **2**.

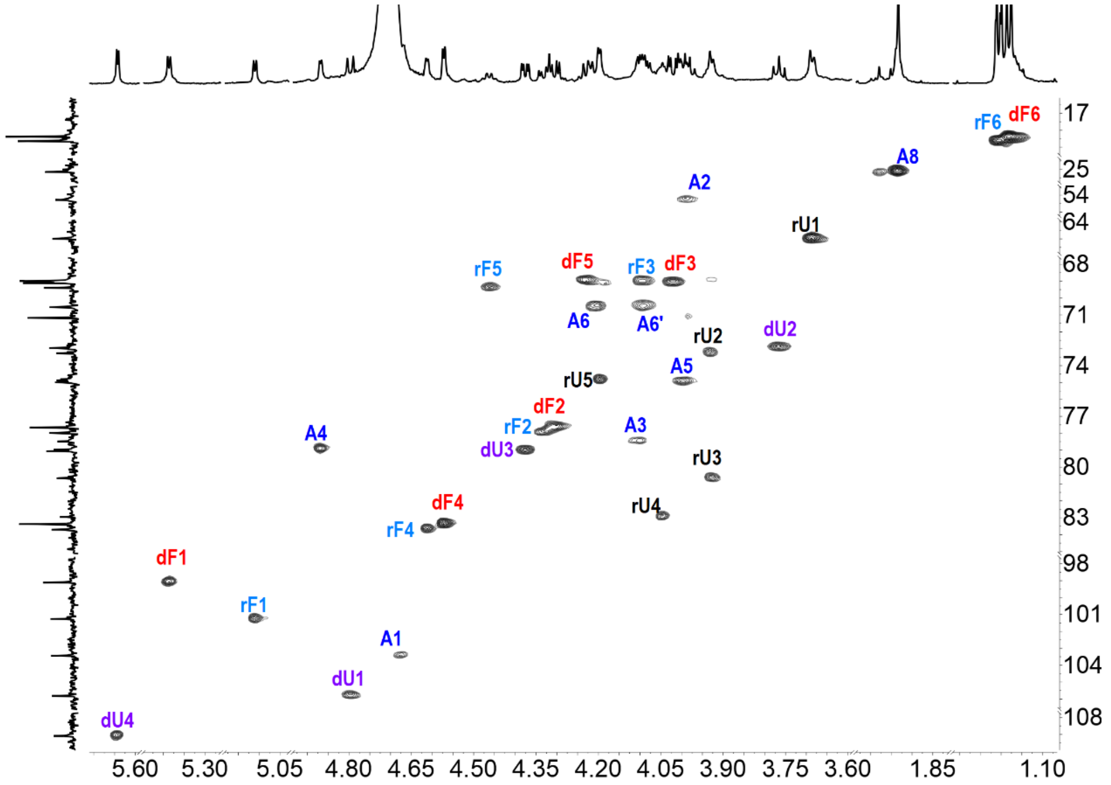

Figure S3. The HSQC spectrum and signal assignments of **2**.

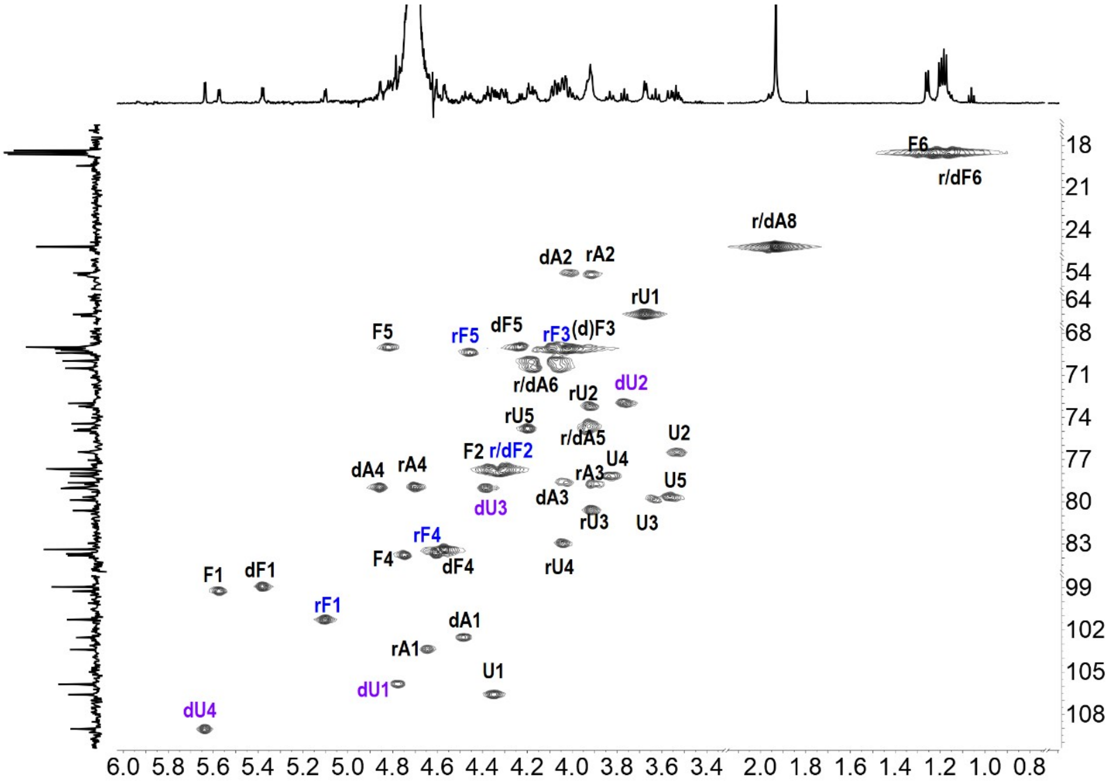

Figure S4. The HSQC spectrum and signal assignments of **3**.

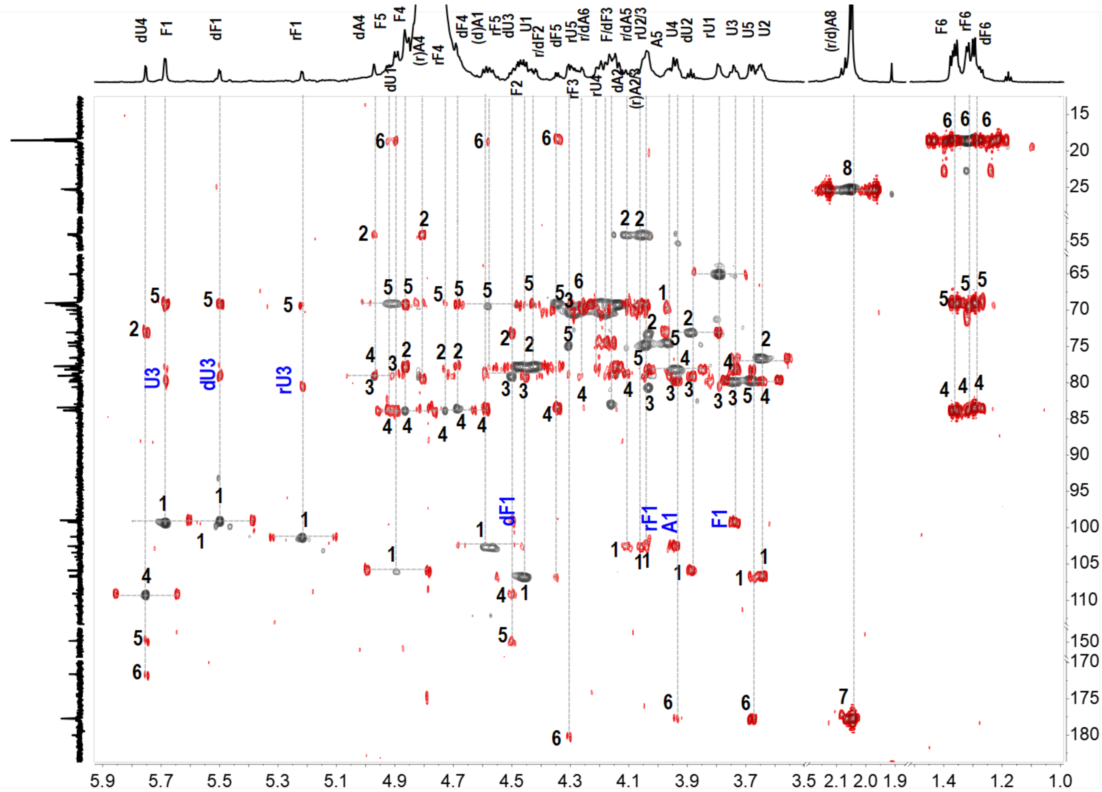

Figure S5. The superimposed ^{1}H–^{13}C correlation NMR spectra (HSQC-gray & HMBC-red) and signal assignments of **4**.

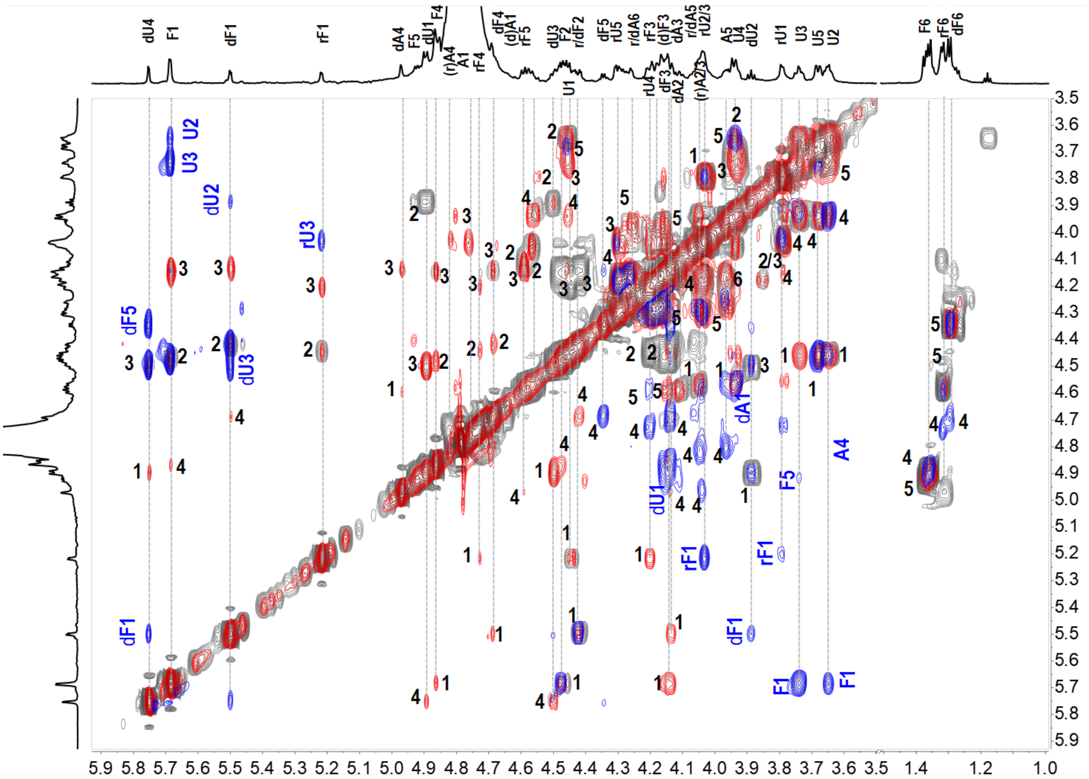

Figure S6. The superimposed ^{1}H–^{1}H correlation NMR spectra (COSY-gray, TOCSY-red, ROESY-blue) and signal assignments of **4**.

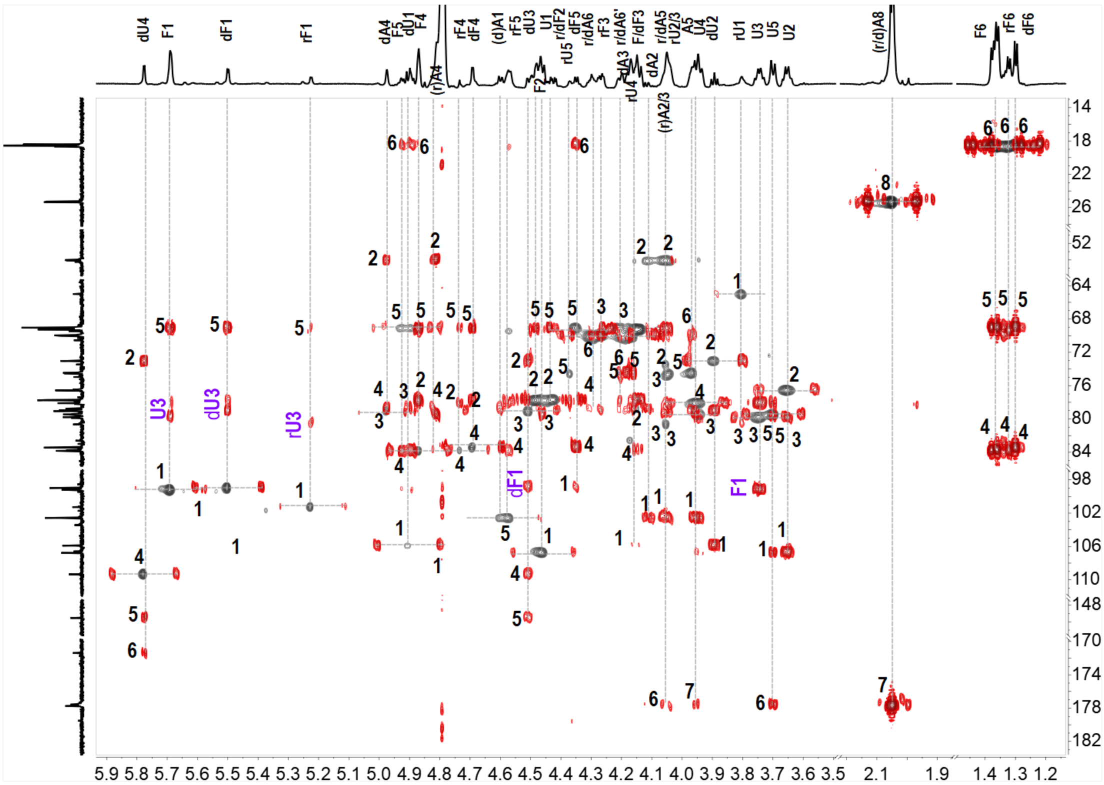

Figure S7. The superimposed ^{1}H–^{13}C correlation NMR spectra (HSQC-gray & HMBC-red) and signal assignments of **5**.

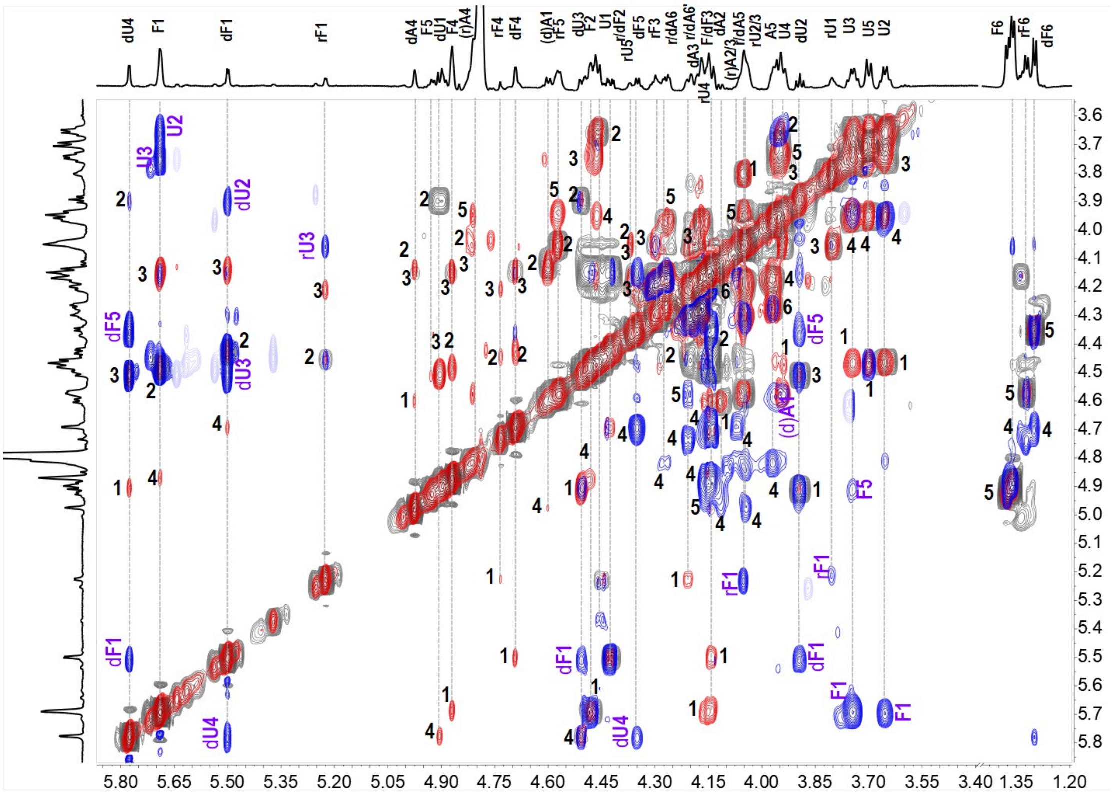

Figure S8. The superimposed ^{1}H–^{1}H correlation NMR spectra (COSY-gray, TOCSY-red, ROESY-blue) and signal assignments of **5**.

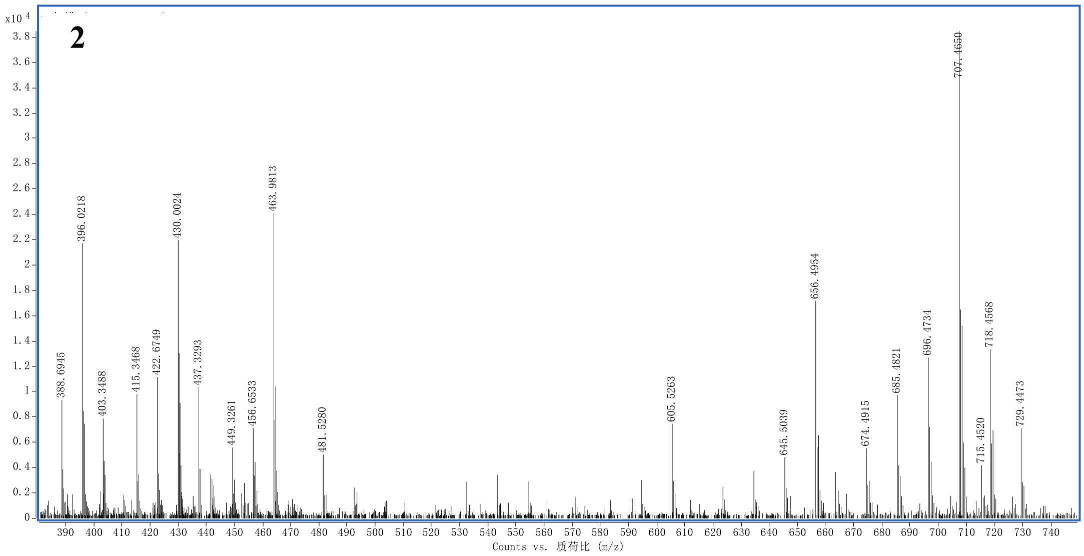
2
x10 4
388.6945
396.0218
403.3488
415.3468
422.6749
430.0024
437.3293
449.3261
456.6533
463.9813
481.5280
605.5263
645.5039
656.4954
674.4915
685.4821
696.4734
707.4650
715.4520
718.4568
729.4473
Counts vs. 质荷比 (m/z)

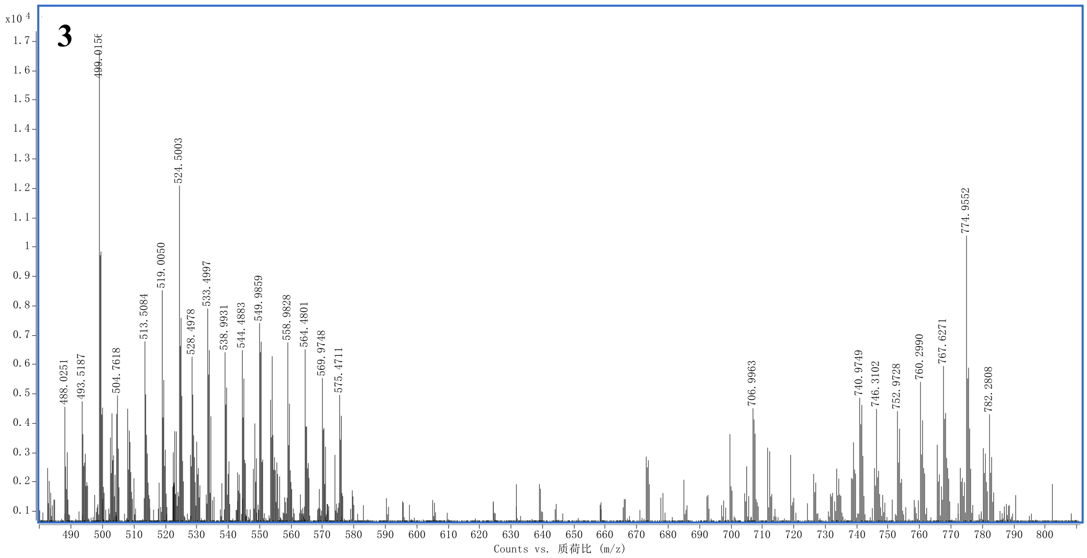
3
x10 4
488.0251
493.5187
499.0156
504.7618
513.5084
519.0050
524.5003
528.4978
533.4997
538.9931
544.4883
549.9859
558.9828
564.4801
569.9748
575.4711
706.9963
740.9749
746.3102
752.9728
760.2990
767.6271
774.9552
782.2808
Counts vs. 质荷比 (m/z)

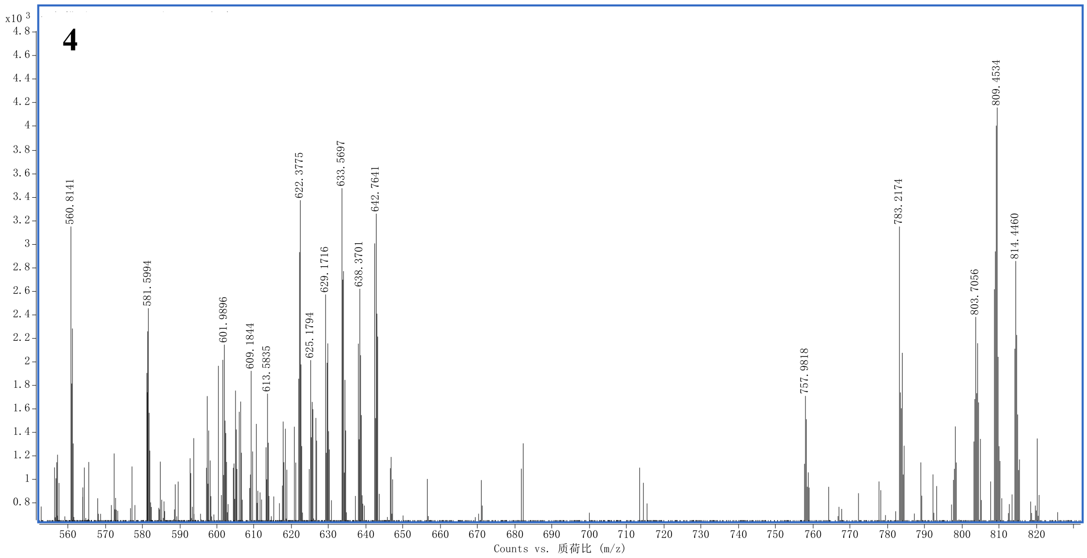
4
x10 3
560.8141
581.5994
601.9896
609.1844
613.5835
622.3775
625.1794
629.1716
633.5697
638.3701
642.7641
757.9818
783.2174
803.7056
809.4534
814.4460
Counts vs. 质荷比 (m/z)

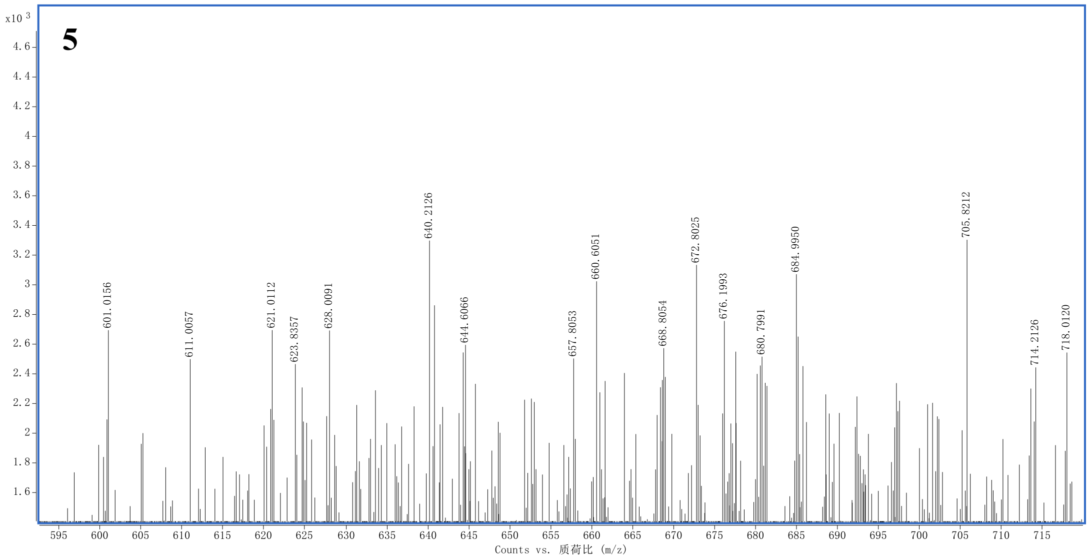

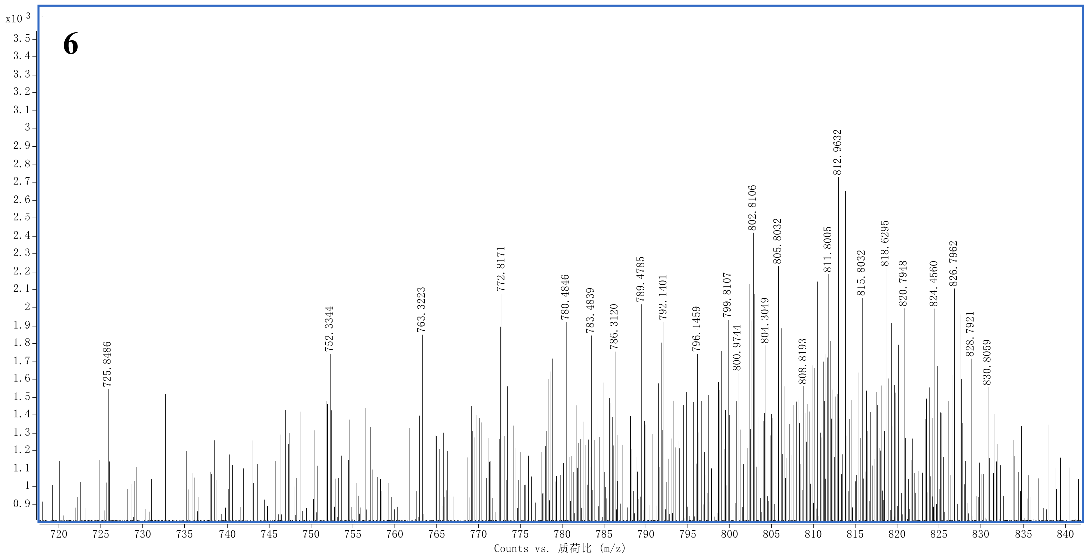

Figure S9. ESI-Q-TOF MS spectra of **2** − **6**.
